# The metagenomic and metabolomic profile of the gut microbes in Chinese full-term and late preterm infants treated with *Clostridium butyricum*

**DOI:** 10.1038/s41598-023-45586-2

**Published:** 2023-10-31

**Authors:** Hong Li, Xingling Ma, Yongfu Li, Qin Liu, Qiuyan Tian, Xiaofeng Yang, Zhemin Zhou, Jing Ren, Bin Sun, Xing Feng, Hong Zhang, Xiaoping Yin, Heng Li, Xin Ding

**Affiliations:** 1 Soochow Key Laboratory of Prevention and Treatment of Child Brain Injury, Children’s Hospital of Soochow University, #303 Jingde Road, Gusu District, Suzhou, 215003 Jiangsu China; 2 Neonatology Department, Suzhou Science and Technology Town Hospital, Suzhou, Jiangsu China; 3 Pediatric Department, Suzhou New District Yangshan Community Health Service Center, Suzhou, China; 4 Pasteurien College, Suzhou Medical College of Soochow University, Suzhou, Jiangsu China; 5 Taixing People’s Hospital, Taizhou, Jiangsu China

**Keywords:** Microbiology, Bacteriology, Clinical microbiology, Microbial communities

## Abstract

The present study investigated the composition, abundance, and diversity of gut microbes in full-term and late-preterm infants from a medical center in eastern China. A total of 144 genomes of stool samples were captured for 16S rRNA metagenomic analyses. A high abundance of commensal intestinal bacteria was detected in these samples such as *Phocaeicola vulgatus, Escherichia coli, and Faecalibacterium prausnitzii*, indicating a relatively consistent diversity of gut microbes in the present full-term infants aged 38–40 weeks. However, late preterm infants (n = 50) with mandatory antimicrobials feeding exhibited lower diversity but a higher composition of opportunistic pathogens such as *Enterococcus* species*.* Centralized on the situation, we explored the regulatory effect of *Clostridium butyricum* as probiotics on these late preterm infants. The consumption of *C. butyricum* did not restore the composition of gut microbes altered by antimicrobials to normal levels, although several opportunistic pathogens decreased significantly after probiotic therapy including *Staphylococcus aureus*, *Sphingomonas echinoides*, and *Pseudomonas putida*. We also compared the effects of day-fed versus night-fed probiotics. Intriguingly, the nighttime feeding showed a higher proportion of *C. butyricum* compared with probiotic day-feeding. Finally, fecal metabolome and metabolites were analyzed in late preterm infants with (n = 20) or without probiotic therapy (n = 20). The KEGG enrichment analysis demonstrated that vitamin digestion and absorption, synaptic vesicle cycle, and biotin metabolism were significantly increased in the probiotic-treated group, while MSEA indicated that a series of metabolism were significantly enriched in probiotic-treated infants including glycerolipid, biotin, and lysine, indicating the complex effects of probiotic therapy on glutathione metabolism and nutrients digestion and absorption in late preterm infants. Overall, this study provided metagenomic and metabolomic profile of the gut microbes in full-term newborns and late preterm infants in eastern China. Further studies are needed to support and elucidate the role of probiotic feeding in late preterm infants with mandatory antimicrobial treatment.

## Introduction

Research over the past decades has revealed an intense link between human health and the resident gut microbes in both adults and children^1^. The gut microbes represent diverse communities of functional microbial species, some of which may play significant roles in energy absorption, nutrient synthesis, and stimulation of the innate and adaptive immune systems, which are highly important for neonatal tolerance and resistance to infection with opportunistic pathogens^2^.

The gut microbial colonization of infants occurs during the period of parturition and gradually develops into a stable adult microbiome by the age of 2 to 3^3^. However, a series of human factors may affect the composition of gut microbes including mode of delivery, type of feeding, and use of antimicrobials^4^. Current research has shown that antimicrobial therapy in late preterm infants can drastically affect the gut microbes and result in population shifts to sole antimicrobial-resistant bacteria such as *Escherichia coli*, *Enterobacter* spp*.*, and *Klebsiella pneumoniae*^5, 6^.

Probiotics are thought to be a viable therapy to prevent newborn necrotizing enterocolitis and sepsis since they provide host health benefits when consumed in sufficient doses^7^. Probiotics such as *Lacticaseibacillus rhamnosus*, *Bifidobacterium animalis* and *Clostridium butyricum* had beneficial effects on the gastrointestinal tract and immune system^8–11^. Specifically, the probiotic *C. butyricum*, identified as the live microorganisms beneficial for the health of the host, was proven to be beneficial by ameliorating the gut bacterial unbalance in children and experimental animals^10, 12–15^. The treatment with probiotics has been shown to impact adult bacterial population structure and functional activity positively^16^.

Additionally, *C. butyricum* regulates the microbes of the digestive tract by boosting the population of lactic acid bacteria and avoiding antibiotic-associated diarrhea^11^. Previous studies indicated that the consumption of *L. rhamnosus* and *B. animalis* in preterm newborns had changed the early colonization of gut bacteria, without further effect on the overall longitudinal bacterial progression during the neonatal period^8, 9^. However, does the probiotic *C. butyricum* have a comparable impact on the gut microbes of newborns? Will intestinal pathogens be affected by the consumption of *C. butyricum*? These problems still require clarification.

In this study, we describe the composition, abundance, and diversity of gut microbes in infants and compare the variance between late preterm and full-term infants via 16S rRNA metagenomic sequencing. We aimed to identify bacterial species that potentially dominate the common gut microbes in infants aged from premature birth to full term. To concentrate on late preterm infants that were mandatorily fed with antimicrobials, a candidate probiotic was applicated to these infants to determine the ability to restore the composition of the gut microbes altered by antimicrobials to its average level.

## Methods and materials

### Ethics approval and consent to participate

The study was approved by the Human Research Ethics Committees of the Children's Hospital of Soochow University (Reference 2020CS017). All specimens were collected according to the guidelines set by the Children's Hospital of Soochow University. All authors confirm that all methods were performed in accordance with the relevant guidelines and regulations (Declaration of Helsinki). Written informed consents were obtained from the parents of all the enrolled infants before specimen collection.

### Subject and selection criteria

The present study aimed to evaluate the composition, abundance, and diversity of gut microbes in Chinese full-term infants and late preterm infants. A total of 44 full-term infants and 50 late preterm infants admitted to the Neonatal Department between November 2020 and April 2021 were enrolled as participants in Suzhou, East China. All the enrolled infants were otherwise healthy without any symptoms of pediatric diseases. The infants’ parents were neither infected nor treated with any antimicrobials.

Detailed infants background and selection criteria could be found in Tables 1, 2, and Supplementary Table 1. The full-term group included 24 girls and 20 boys with gestational ages ranging from 37 to 40 + 5 weeks and birth dates from the first day of life to the 12th day. They are given a mixture of breast milk and formula milk and have an average birth weight of 3152 g.

**Table 1:** Groups design and description.

Groups design	Groups description*	Numbers
Group A	Full-term infants aged 37–40 weeks	N = 44
Group B	Late preterm infants one week after premature birth	N = 50
Group C	Late preterm infants with probiotic therapy	N = 50
Group C_D (Day)	Late preterm infants fed with probiotic in daytime	N = 25
Group C_N (Night)	Late preterm infants fed with probiotic in nighttime	N = 25

*The term infants are breastfed, while special formula milk for preterm neonates was given.

**Table 2:** Selection criteria for study subjects.

	Criterion
1	The full-term infant was 37–40 weeks after cesarean or vaginal birth at the time of study
2	Free of intrauterine asphyxia, with an Apgar score > 7/min and stable vital signs within 24 h after birth
3	Free of complication, such as severe infections, cardiopulmonary diseases, congenital genetic diseases, or gastrointestinal malformation
4	Hospital stay ≥ 7 days
5	No infusion of hormones or blood products during hospitalization, except for albumin
6	The mother had no history of infection during pregnancy or long-term use of antimicrobials, hormones, or blood products
7	While hospitalized, latamoxef was the only acceptable antimicrobial medication, special formula milk for preterm neonates was given, and other nutritional supplementation was administered intravenously
8	Full-term infants did not receive any antimicrobial therapies during the hospital stay

The late-preterm group included 24 girls and 26 boys with gestational ages ranging from 33 to 36 + 6 weeks and a birth age ranging from 1 to 7 days. There were 21 vaginal births and 29 cesarean births. All were fed Nestle preterm baby milk and had an average birth weight of 2329.2 g. Stool samples were collected from the late-preterm group before probiotic treatment.

Then, the late-preterm newborns were provided probiotics and subsequently placed into two groups: day feeding and evening feeding. The day-feeding group included 12 female newborns and 13 male infants with gestational ages ranging from 33 + 4 to 36 + 2 weeks, with a minimum age of one day and a maximum age of seven days. There were 12 vaginal births and 13 cesarean deliveries. The average birth weight was 2338.4 g.

All the mothers had no history of infection during pregnancy or long-term use of antimicrobials, hormones, or blood products. All infants were free of complications, such as severe infections, cardiopulmonary diseases, congenital genetic diseases, or gastrointestinal malformation. While hospitalized, latamoxef was the only acceptable antimicrobial medication. The term infants are breastfed, while special formula milk for preterm neonates was given, and another nutritional supplementation was administered intravenously.

### Group and subgroup

**Figure 1:**
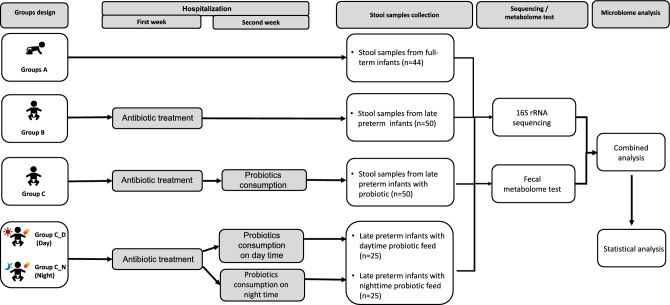
Study workflow. A total of 144 genomes were enrolled for 16S rRNA metagenomic analyses and fecal metabolome analysis.

The full-term infants were clustered as Group A (n = 44) whiles late preterm infants were defined as Group B (n = 50). In addition, the 50 late preterm infants were further treated for *C. butyricum* therapy and stool samples were obtained as Group C. After that the infants from Group C were divided into sub-groups of day-fed *C. butyricum* (Group C_D, C1-C25, n = 25) and night-fed *C. butyricum* (Group C_N, C26-C50, n = 25). Figure 1 displays the detailed workflow. Latamoxef was given intravenously after being calculated using the norm of 40 mg/kg/per according to the weight of late-preterm newborns twice a day. The date of Latamoxef consumption started between November 2020 and June 2021, and the average duration of antibiotic treatment was 12 to 13 days. The probiotic of *C. butyricum* MIYARI 588 was lyophilized powder purchased from Miyarisan Pharmaceutical Co., Ltd. The CFU of *C. butyricum* is 1X10^9^ per gram. Each newborn was given 500 mL of milk with 40 mg of *C. butyricum* MIYARI 588 twice a day at 8 a.m. and 3 p.m. for the day group, and 8 p.m. and 3 a.m., for night group, with feces collected one week later.

### Sample

For Group A, the full-term infants were fed without antibiotics or probiotics, and the stool collection was the first stool in hospitalization within 24h, with a collection date between November 13, 2020, and May 21, 2021. For Group B, antibiotics were administered to premature babies hospitalized between October 27, 2020, and June 4, 2021. Feces were collected from preterm newborns between November 2, 2020, and June 7, 2021, without any probiotics consumed. For Group C, probiotics were first given to the preterm infants between November 2, 2020, and June 7, 2021. After probiotic feeding for one week, feces were collected from those infants between November 9, 2020 to June 14, 2021.Additionally, the stool samples were split into groups that received probiotics during the day and those who received them at night, depending on the feeding schedule. Then, each specimen was about 5 g in weight, gathered using a sterile disposable stool sampler and kept in a – 80 ℃ refrigerator for subsequent 16S rRNA sequencing to analyze the composition, abundance, and diversity of the gut microbes. Aseptic precautions were exercised during all procedures.

### DNA extraction and 16S rRNA sequencing

After being removed from the sampler, the stool was diluted 5X with molecular grade water and homogenized with a vortex. The stool suspension (250 µl) was used for DNA extraction. The genomic DNA was purified and isolated with MagaBio Soil/Feces Genomic DNA Purification Kit (Hangzhou Bioer Technology, Hangzhou, Zhejiang, China). The extracted DNA was quantified at an A260/A280 nm ratio with the NanoDrop ND-1000 (Thermo Fisher Scientific, Cleveland, OH, USA). Then extraction from each sample was diluted to approximately 5 ng/µl and stored at – 20 °C for 16S rRNA sequencing.

The 12-bp barcoded primers synthesized by Invitrogen (Invitrogen, Carlsbad, CA, USA) were used to amplify the bacterial 16S rRNAV3-V4 fragments. The PCR mixture contained 25 μl reactions Taq (Takara Biotechnology, Dalian, Liaoning, China), 1 μl of each primer (10 mM), and 3 μl DNA (20 ng/μl) template (final volume: 50 μl). The PCR protocol was as follows: 94 °C for 30 s followed by 30 cycles of denaturation at 94 °C for 30 s, annealing at 52 °C for 30 s, and extension at 72 °C for 30 s; followed by a final extension at 72 °C for 10 min. The PCR products were subjected to 1% agarose gel electrophoresis and then sequenced on an Illumina Miseq (PE 300) in the MAGIGENE Genomic Institute. The PCR products were mixed according to the Instructions of the GeneTools Analysis Software (Version 4.03.05.0, SynGene).

### Bioinformatic analyses of genomic data

Pair-end reads for each sample were merged using the BBmerge module in the BBtools suite v38.94 with options of “loose = t mininsert = 120 mininsert0 = 100 qtrim2 = t qout = 33 entropy = t maxns = 0 trimq = 10”. Low-quality sequences in the merged DNA fragments were removed using the “fastq_filter” module in Vsearch v2.18.0 with a fastq_maxee_rate of 0.02. The “cluster” module in the Vsearch package was applied to the remaining set of high-quality sequences to generate clusters of nearly identical sequences (identities ≥ 0.997) and extract one centroid sequence for each cluster. The same package was applied to remove potential sequencing errors and chimeric sequences from the centroid sequences using the “cluster_unoise” module and the “uchime3_denovo” module, respectively^17^. The resulting set of non-repetitive, high-quality sequences was treated as ASVs (Amplicon sequence variants) and subjected to taxonomic assignment based on their comparison results with the SILVA database of 16S rRNA (release 138.1; https://www.arb-silva.de/) using BLASTn. We kept hits that shared ≥ 85% identities in ≥ 50% of the sequences of the reads. The final taxonomic information of each read was determined based on hybrid LCA criteria^18^.

The Quantitative Insights in Microbial Ecology (QIIME) software (Version 1.9.1) was enrolled to calculate Observed-otus, Chao1, Shannon, Simpson, ace, Goods-coverage, and PD_whole_tree index^19^. R software (Version 4.1.2) was applied to draw dilution curve, Rank abundance curve, species accumulation curve and analyze the difference between groups of Alpha diversity index. The used T-test and wilcox test were used to analyze the difference between groups of Alpha diversity index. Specifically, the Chao1 estimator and the ACE estimator were used to calculate community richness, while the Shannon index and the Simpson index were applied in community diversity analysis (http://scikit-bio.org/docs/latest/generated/skbio.diversity.alpha). R software (Version 4.1.2) was used to plot PCA, PCoA and NMDS plots. The stats package and ggplot2 package of R software was used for PCA and PCoA analysis, and the vegan package of R software was used for NMDS analysis. Use R software to analyze the differences between groups of Beta diversity index and perform parametric test and non-parametric test respectively. Stamp analysis was performed using Stamp software, and the filter value of Score was set to default. Metastats analysis uses Mothur software at each classification level (Phylum, Class, Order, Family, Genus, Species) to perform a permutation test between groups to obtain a p-value, and then use the Benjamini and Hochberg False Discovery Rate method to correct the p-value. Anosim, MRPP and Adonis analysis use the anosim function, mrpp function and adonis function of the R vegan package, respectively. AMOVA analysis was performed using the above function of the mothur software. Species with significant differences between groups were analyzed using R software for T-test between groups and plotted.

### Fecal metabolomes evaluation

Background of the 40 late-preterm infants enrolled in metabolome analysis was showed in Table 4. The sample stored at − 80 °C refrigerator was thawed on ice. A 400 μL solution (Methanol:Water = 7:3, V/V) containing internal standard was added into 20 mg sample, and vortexed for 3 min. The sample was sonicated in an ice bath for 10 min and vortexed for 1 min, and then placed at − 20 °C for 30 min. The sample was then centrifuged at 12,000 rpm for 10 min (4 °C). And the sediment was removed, then centrifuged the supernatant was at 12,000 rpm for 3 min (4 °C). A 200 μL aliquots of supernatant were transferred for LC–MS analysis^20^.

Detail LC–MS method was described as follows^21, 22^. The analytical conditions of LC–MS were as follows, UPLC: column, Waters ACQUITY UPLC HSS T3 C18 (1.8 um, 2.1 mm*100 mm); column temperature, 40 °C; flow rate, 0.4 mL/min; injection volume, 2 μL; solvent system, water (0.1% formic acid): acetonitrile (0.1% formic acid); The column was eluted with 5% mobile phase B (0.1% formic acid in acetonitrile) at 0 min followed by a linear gradient to 90% mobile phase B (0.1% formic acid in acetonitrile) over 11 min, held for 1 min, and then come back to 5% mobile phase B within 0.1 min, held for 1.9 min. The data acquisition was operated using the information-dependent acquisition (IDA) mode using Analyst TF 1.7.1 Software (Sciex, Concord, ON, Canada). The source parameters were set as follows: ion source gas 1 (GAS1), 50 psi; ion source gas 2 (GAS2), 50 psi; curtain gas (CUR), 35 psi; temperature (TEM), 550 °C, or 450 °C; declustering potential (DP), 60 V, or-60 V in positive or negative modes, respectively; and ion spray voltagefloating (ISVF), 5500 V or-4500 V in positive or negative modes, respectively. The TOF MS scan parameters were set as follows: mass range, 50–1000 Da; accumulation time, 200 ms; and dynamic background subtract, on. The product ion scan parameters were set as follows: mass range, 25–1000 Da; accumulation time, 50 ms; collision energy, 30 or-30 V in positive or negative modes, respectively; collision energy spread, 15; resolution, UNIT; charge state, 1 to 1; intensity, 100 cps; exclude isotopes within 4 Da; mass tolerance, 50 mDa; maximum number of candidate ions to monitor per cycle.

All samples were acquired by the LC–MS system machine orders in Metware company, China. The original data file acquisition by LC–MS was converted into mzML format by ProteoWizard software. Peak extraction, peak alignment and retention time correction were respectively performed by XCMS program. The “SVR” method was used to correct the peak area. The peaks with detection rate lower than 50% in each group of samples were discarded. After that, metabolic identification information was obtained by searching the laboratory’s self-built database, integrated public database, AI database and metDNA. For two-group analysis, differential metabolites were determined by VIP (VIP > 1) and P-value (P-value < 0.05, paired t-test). VIP values were extracted from OPLS-DA result, which also contains score plots and permutation plots, and was generated using R package MetaboAnalystR v1.0.1. The data was log transform (log2) and mean centering before OPLS-DA. Identified metabolites were annotated using KEGG Compound database (http://www.kegg.jp/kegg/compound/), and annotated metabolites were then mapped to KEGG Pathway database (http://www.kegg.jp/kegg/pathway.html). Significantly enriched pathways are identified with a hypergeometric test’s P-value for a given list of metabolites. The compound name of internal standard used in LC/MS analysis were described in Supplementary Table 2.

### Statistical analysis

The analysis of diversity, variance and similarity with repeated measures was used to determine the statistical significance for each experimental group. Statistical analyses were carried out in R (4.0.3). Vegan (v 2.5–6) was used for all alpha-diversity calculations: index of Shannon, Simpson, Chao1, and ACE diversity (alpha diversity measurement of evenness and richness); evenness (homogeneous the distribution of taxa counts) and richness (number of taxa in a community). Pairwise Bray–Curtis dissimilarity was used to assess beta-diversity, or the overall variation between each sample^23^. The Bray–Curtis dissimilarity metric compares two communities based on the number or relative abundance of each taxon present in at least one of the communities. The differential metabolites were determined by VIP (VIP > 1) and P-value (P-value < 0.05, paired t-test). Analyses were conducted using GraphPad Prism (v7) with statistical significance accepted as *P < 0.05, **P < 0.01, ***P < 0.005, ****P < 0.001. The images of Barplot, PCA, PcoA, and Stamp were created on Tutools platform (http://www.cloudtutu.com). The ANOSIM analysis, Violin and Heatmap were plotted by http://www.bioinformatics.com.cn, a free online platform for data analysis and visualization. Statistical analysis of differential metabolites and MSEA enrichment annotated by KEGG pathways were displayed in Supplementary Tables 3 and 4.

## Results

### High abundance of commensal intestinal bacteria was detected in full-term infants

**Figure 2:**
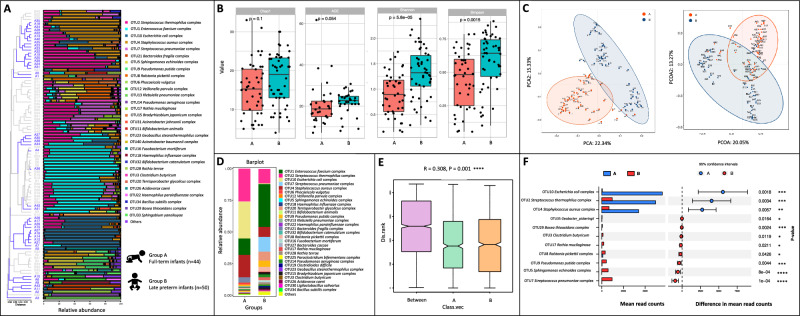
The composition, abundance and diversity of gut microbes in full-term infants (n = 44) and late preterm infants (n = 50). (**A**) Barplot of bacterial composition and relative abundance. (**B**) Alpha diversity of gut microbes. Chao1, Ace, Shannon, and Simpson indexes are presented. (**C**) PCA and PcoA analyses of Beta diversity. (**D**) General bacterial composition and relative abundance of the groups. (**E**) Analysis of similarities (ANOSIM). The y-axis represented the interquartile range, and the index of between reflected the difference between groups. (**F**) Stamp variation analysis indicated the difference in bacterial genus between groups.

The overall process of this study is in Fig. 1. Initially, we characterized the gut microbes among the full-term infants aged 38–40 weeks (Group A). A high abundance of commensal intestinal bacteria was detected in these samples such as *Streptococcus thermophilus*, *Phocaeicola vulgatus, Escherichia coli, and Faecalibacterium prausnitzii*, indicating a relatively consistent diversity of gut microbes in the present full-term infants aged 38–40 weeks (Fig. 2A).

### Opportunistic pathogens occupied and colonized late preterm infants

We subsequently compare the gut microbes between the full-term infants (Group A) and late preterm infants aged only one week after premature birth (Group B). At the overall level of gut microbes, no significance was detected in α diversity corresponding to community richness (Fig. 2B, estimator of Chao1 and ACE), However, the Shannon and Simpson index showed that full-term newborns had less community diversity than late-preterm infants, with commensal *Escherichia coli* being the predominant bacterium (Fig. 2B). In addition, β diversity, which reflected the differences in microbial communities between samples, was calculated and visualized in PCA, PCoA (Fig. 2C) and ANOSIM (Fig. 2E). Compositional variation targeting specific bacteria was observed among groups, and opportunistic pathogens were found to occupy and colonize late preterm infants, including *Enterococcus faecium* and other strictly aerobic bacteria (Fig. 2F). Notably, a minority of oral bacteria was detected in late preterm infants such as *Veillonella parvula*, *Haemophilus influenzae*, *Rothia mucilaginosa,* and *Streptococcus sanguinis* (Fig. 2A,D).

### Probiotic therapy declined the abundance of pathogenic microbes in late preterm infants

**Figure 3:**
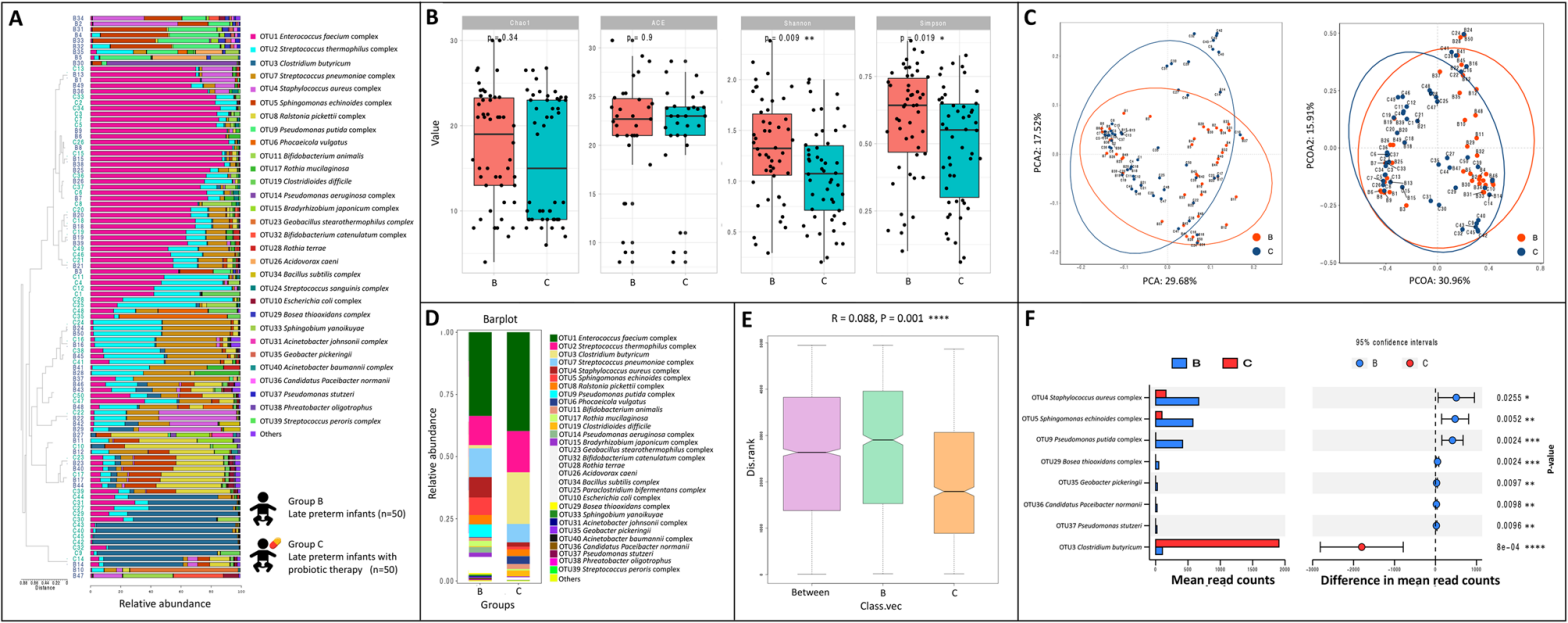
The composition, abundance and diversity of gut microbes in late preterm infants with probiotic therapy (n = 50) or without (n = 50). (**A**) Barplot of bacterial composition and relative abundance. (**B**) Alpha diversity of gut microbes. Chao1, Ace, Shannon, and Simpson indexes are presented. (**C**) PCA and PcoA analyses of Beta diversity. (**D**) General bacterial composition and relative abundance of the groups. (**E**) Analysis of similarities (ANOSIM). The y-axis represented the interquartile range, and the index of between reflected the difference between groups. (**F**) Stamp variation analysis indicated the difference in bacterial genus between groups.

Afterward, probiotic therapy was administered in the late preterm infants by consumption of *C. butyricum* (Group B and C), which became detectable in the majority of the late preterm infants (Fig. 3A–F). However, there were no differences in community richness whiles significance was observed in community diversity by Shannon and Simpson index (Fig. 3B). Intriguingly, several opportunistic pathogens were found to decline significantly after probiotic therapy including *Staphylococcus aureus*, *Sphingomonas echinoides,* and *Pseudomonas putida*, indicating a reduction in community diversity in these probiotic-fed neonates.

**Figure 4:**
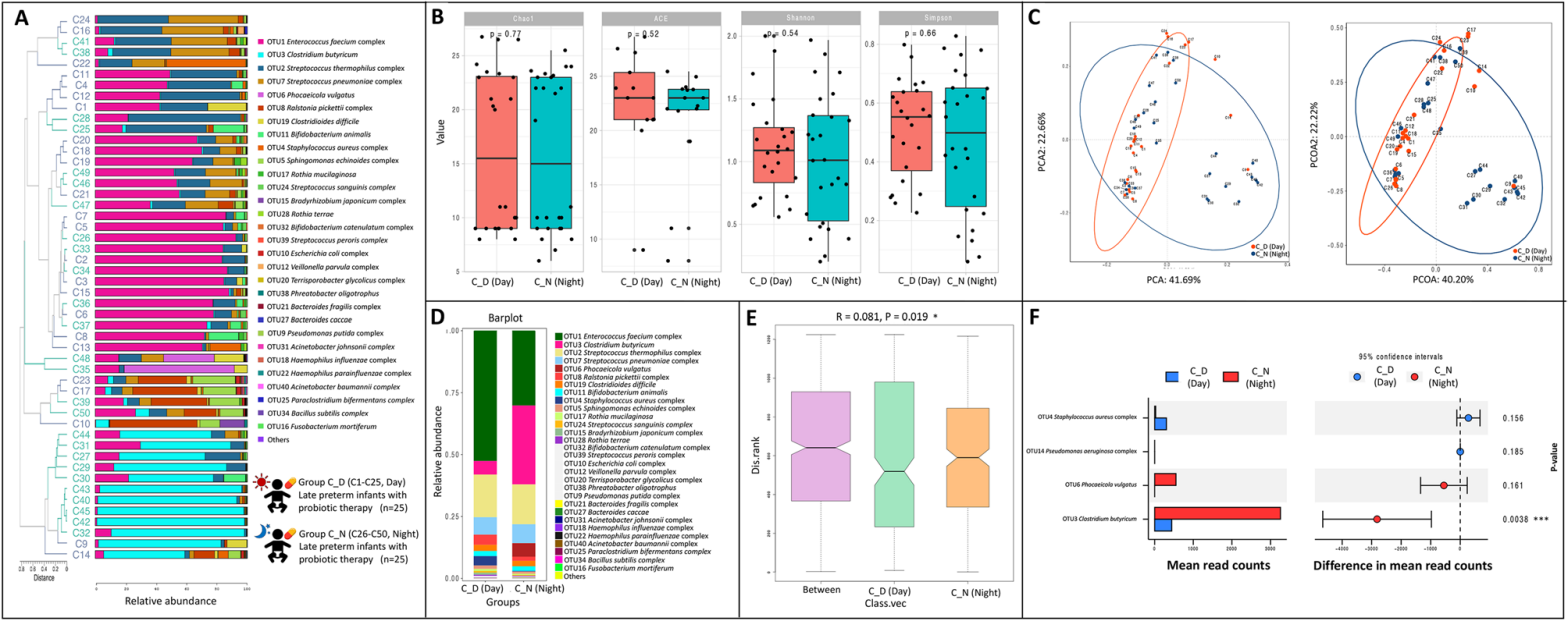
The composition, abundance and diversity of gut microbes in late preterm infants with probiotic therapy in daytime (n = 25) or at night (n = 25). (**A**) Barplot of bacterial composition and relative abundance. (**B**) Alpha diversity of gut microbes. Chao1, Ace, Shannon, and Simpson indexes are presented. (**C**) PCA and PcoA analyses of Beta diversity. (**D**) General bacterial composition and relative abundance of the groups. (**E**) Analysis of similarities (ANOSIM). The y-axis represented the interquartile range, the index of between reflected the difference between groups. (**F**) Stamp variation analysis indicated the difference in bacterial genus between groups.

**Figure 5:**
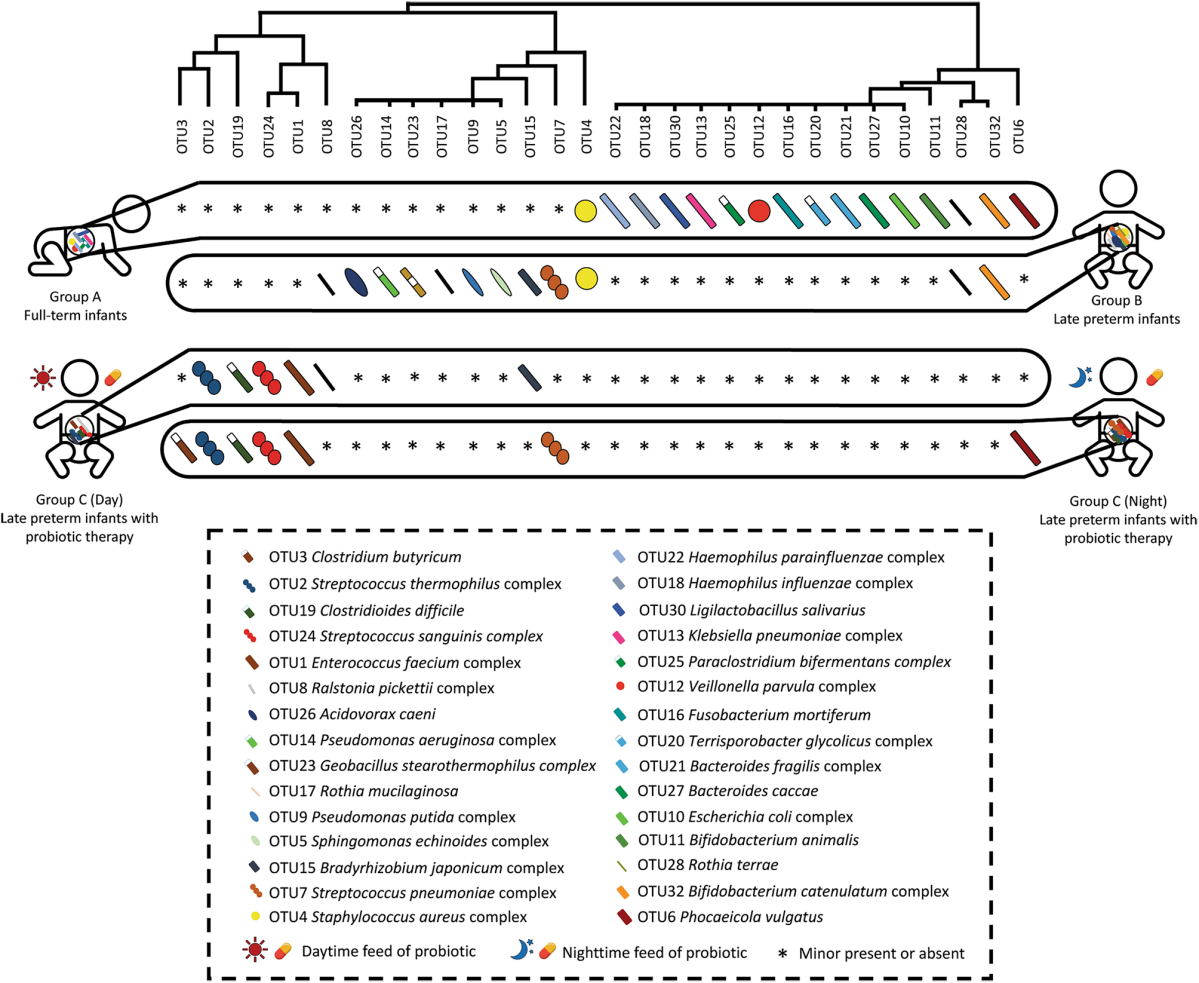
Overview of the top 30 abundance of bacterial genera/species of gut microbes in full-term infants and late preterm infants. The abundance was calculated according to the results of all 144 stool samples. Asterisks indicate that the genus were not the dominant bacteria in the gut of the infants.

Finally, we compared the effects of day-fed (Group C_D) versus night-fed (Group C_N) probiotics (Fig. 4A–F). We noted a decrease of *Enterococcus faecium* in night-fed compared with day-fed. Intriguingly, the nighttime feeding showed a higher proportion of *C. butyricum* (Fig. 4F, p = 0.0038). However, this study showed that the consumption of *C. butyricum* did not restored the composition of gut microbes altered by antimicrobial agents to normal levels (Fig. 5).

### Effects of probiotic therapy on fecal metabolome and metabolites in late preterm infants

To further illustrate the effects of probiotic therapy on fecal metabolome and metabolites in late preterm infants, all 144 fecal samples were sent for untargeted metabolomics analysis. After quality control, a number of 40 fecal samples passed the criteria. Specifically, there were 20 samples from the probiotic therapy group and 20 samples without any probiotics received. The average gestational ages were 34.6 weeks for both groups, while average ages were 4.05 days and 3.88 days for probiotic therapy group and control group, respectively. The detailed background of enrolled infants was showed in Tables 3 and 4. The compound name of internal standard used in LC/MS analysis was described in Supplementary Table 2.

**Table 3:** Background of the full-term and late-preterm infants enrolled in this study.

Group	Infants	Gender	Average gestational age (weeks)	Average age (days)	Mode of delivery #	Average initial birth weight (g)	Date of hospitalization	Antibiotics	Date of antibiotics consumption	Date to stop antibiotics	Average duration of antibiotic treatment (days) ^	Probiotics	Date of probiotic consumption	Time of probiotics consumption *	Date of 1st stool	Date of 2nd stool	History of infection
A	full-term infants (44)	female (24), male (20)	38	3	VD (44)	3153	Nov 2020 to Mar 2021	/	/	/	/	/	/	/	Nov 2020 to May 2021	None	None
B	late preterm infants (50)	female (24), male (26)	35	4	VD (21), CS (29)	2329	Nov 2020 to Jun 2021	latamoxef	Nov 2020 to Jun 2021	Nov 2020 to Jun 2021	13	/	/	/	Nov 2020 to Jun 2021	None	None
C	late preterm infants with probiotic (50)	female (24), male (26)	35	4	VD (21), CS (29)	2329	Oct 2020 to Jun 2021	latamoxef	Nov 2020 to Jun 2021	Nov 2020 to Jun 2021	13	*Clostridium Butyricum* Powders	Nov 2020 to Jun 2021	Mix consumptions	/	Nov 2020 to Jun 2021	None
C_D	Late preterm infants with daytime probiotic feed (25)	female (12), male (13)	37	3	VD (9), CS (16)	3115	Oct 2020 to Jun 2021	latamoxef	Oct 2020 to Jun 2021	Nov 2020 to Jun 2021	12	*Clostridium Butyricum* Powders	Nov 2020 to Jun 2021	8 a.m. ~ 3 p.m. (25)	/	Nov 2020 to Jun 2021	None
C_N	Late preterm infants with nighttime probiotic feed (25)	female (12), male (13)	37	3	VD (12), CS (13)	3115	Oct 2020 to May 2021	latamoxef	Oct 2020 to May 2021	Nov 2020 to Jun 2021	13	*Clostridium Butyricum* Powders	Nov 2020 to May 2021	8 p.m. ~ 3 a.m. (25)	/	Nov 2020 to Jun 2021	None

^#^VD, vaginal delivery, CS, cesarean section.

^Indicates the average duration of antibiotics treatment.

*Mix consumptions indicates the feeding of both daytime (8a.m. ~ 3p.m.) and nighttime (8p.m. ~ 3a.m.).

**Table 4:** Background of the 40 late-preterm infants enrolled in metabolome analysis.

Groups	Gender	Gestational age/weeks	Age/day	Mode of delivery	Initial birth weight (g)	Date of hospitalization (YY/MM/DD)	Antibiotics	Date of antibiotics consumption (YY/MM/DD)	Date to stop antibiotics (YY/MM/DD)	Duration of antibiotics treatment	Probiotics	Date of probiotics consumption (YY/MM/DD)	Time of probiotics consumption	Sampling date (YY/MM/DD)	Metabolome analysis
late preterm infants (n = 20)	Female	36 + 1	3	VD	2770	2021/5/24	latamoxef	2021/5/25	2021/6/6	12 days	/	/	/	2021/5/26	Yes
	Male	33 + 6	6	CS	1910	2020/10/27	latamoxef	2020/10/27	2020/11/12	16 days	/	/	/	2020/11/2	Yes
	Male	35 + 5	7	CS	2460	2020/11/2	latamoxef	2020/11/2	2020/11/12	10 days	/	/	/	2020/11/8	Yes
	Male	35 + 2	5	CS	1950	2021/11/11	latamoxef	2020/11/11	2020/12/2	21 days	/	/	/	2020/11/16	Yes
	Male	34 + 5	3	VD	2840	2020/11/13	latamoxef	2020/11/13	2020/11/26	13 days	/	/	/	2020/11/16	Yes
	Female	35 + 1	3	VD	1930	2020/11/22	latamoxef	2020/11/22	2020/12/6	14 days	/	/	/	2020/11/23	Yes
	Female	35	3	CS	2590	2020/12/1	latamoxef	2020/12/1	2020/12/14	13 days	/	/	/	2020/12/4	Yes
	Female	34 + 3	3	VD	2180	2020/12/1	latamoxef	2020/12/2	2020/12/22	20 days	/	/	/	2020/12/4	Yes
	Male	36 + 1	4	CS	1980	2020/12/24	latamoxef	2020/12/24	2021/1/5	12 days	/	/	/	2020/12/28	Yes
	Female	33	4	VD	1900	2021/1/2	latamoxef	2021/1/1	2021/1/8	7 days	/	/	/	2021/1/5	Yes
	Male	35 + 3	1	VD	2620	2021/12/29	latamoxef	2020/12/29	2021/1/6	8 days	/	/	/	2021/1/5	Yes
	Female	33 + 5	2	VD	2630	2021/11/13	latamoxef	2021/1/13	2021/1/21	8 days	/	/	/	2021/1/15	Yes
	Male	36 + 6	7	VD	2480	2021/1/18	latamoxef	2021/1/19	2021/1/22	3 days	/	/	/	2021/1/20	Yes
	Male	35 + 6	1	VD	2790	2021/1/21	latamoxef	2021/1/21	2021/1/29	8 days	/	/	/	2021/1/22	Yes
	Male	33 + 6	7	CS	2500	2020/10/28	latamoxef	2020/10/29	2020/11/11	13 days	/	/	/	2020/11/2	Yes
	Female	36	6	VD	2250	2020/11/3	latamoxef	2020/11/3	2020/11/12	9 days	/	/	/	2020/11/8	Yes
	Male	33 + 4	5	VD	1950	2021/11/11	latamoxef	2020/11/11	2020/11/23	12 days	/	/	/	2020/11/16	Yes
	Male	35 + 2	5	CS	2550	2020/11/11	latamoxef	2020/11/11	2020/12/2	21 days	/	/	/	2020/11/16	Yes
	Male	35 + 1	3	VD	2120	2020/11/22	latamoxef	2020/11/22	2020/12/6	14 days	/	/	/	2020/11/23	Yes
	Male	34 + 3	3	VD	1700	2020/12/1	latamoxef	2020/12/1	2020/12/19	18 days	/	/	/	2020/12/3	Yes
late preterm infants with probiotic (n = 20)	Female	36 + 1	3	VD	2770	2021/5/24	latamoxef	2021/5/25	2021/6/6	12 days	*C. butyricum* Powders	2021/5/26	8 p.m. 3 a.m	2021/6/2	Yes
	Male	33 + 6	6	CS	1910	2020/10/27	latamoxef	2020/10/27	2020/11/12	16 days	*C. butyricum* Powders	2020/11/3	8 p.m. 3 a.m	2020/11/10	Yes
	Male	35 + 5	7	CS	2460	2020/11/2	latamoxef	2020/11/2	2020/11/12	10 days	*C. butyricum* Powders	2020/11/9	8 p.m. 3 a.m	2020/11/16	Yes
	Male	35 + 2	5	CS	1950	2021/11/11	latamoxef	2020/11/11	2020/12/2	21 days	*C. butyricum* Powders	2020/11/16	8 p.m. 3 a.m	2020/11/23	Yes
	Male	34 + 5	3	VD	2840	2020/11/13	latamoxef	2020/11/13	2020/11/26	13 days	*C. butyricum* Powders	2020/11/16	8 p.m. 3 a.m	2020/11/23	Yes
	Female	35 + 1	3	VD	1930	2020/11/22	latamoxef	2020/11/22	2020/12/6	14 days	*C. butyricum* Powders	2020/11/25	8 p.m. 3 a.m	2020/12/2	Yes
	Female	35	3	CS	2590	2020/12/1	latamoxef	2020/12/1	2020/12/14	13 days	*C. butyricum* Powders	2020/12/4	8 p.m. 3 a.m	2020/12/11	Yes
	Female	34 + 3	3	VD	2180	2020/12/1	latamoxef	2020/12/2	2020/12/22	20 days	*C. butyricum* Powders	2020/12/4	8 p.m. 3 a.m	2020/12/11	Yes
	Male	36 + 1	4	CS	1980	2020/12/24	latamoxef	2020/12/24	2021/1/5	12 days	*C. butyricum* Powders	2020/12/29	8 p.m. 3 a.m	2021/1/5	Yes
	Female	33	4	VD	1900	2021/1/2	latamoxef	2021/1/1	2021/1/8	7 days	*C. butyricum* Powders	2021/1/5	8 p.m. 3 a.m	2021/1/12	Yes
	Male	35 + 3	1	VD	2620	2021/12/29	latamoxef	2020/12/29	2021/1/6	8 days	*C. butyricum* Powders	2021/1/5	8 p.m. 3 a.m	2021/1/12	Yes
	Female	33 + 5	2	VD	2630	2021/11/13	latamoxef	2021/1/13	2021/1/21	8 days	*C. butyricum* Powders	2021/1/19	8 p.m. 3 a.m	2021/1/26	Yes
	Male	36 + 6	7	VD	2480	2021/1/18	latamoxef	2021/1/19	2021/1/22	3 days	*C. butyricum* Powders	2021/1/20	8 p.m. 3 a.m	2021/1/27	Yes
	Male	35 + 6	1	VD	2790	2021/1/21	latamoxef	2021/1/21	2021/1/29	8 days	*C. butyricum* Powders	2021/1/22	8 p.m. 3 a.m	2021/1/29	Yes
	Male	33 + 6	7	CS	2500	2020/10/28	latamoxef	2020/10/29	2020/11/11	13 days	*C. butyricum* Powders	2020/11/2	8 a.m. 3 p.m	2020/11/9	Yes
	Female	36	6	VD	2250	2020/11/3	latamoxef	2020/11/3	2020/11/12	9 days	*C. butyricum* Powders	2020/11/9	8 a.m. 3 p.m	2020/11/16	Yes
	Male	33 + 4	5	VD	1950	2021/11/11	latamoxef	2020/11/11	2020/11/23	12 days	*C. butyricum* Powders	2020/11/16	8 a.m. 3 p.m	2020/11/23	Yes
	Male	35 + 2	5	CS	2550	2020/11/11	latamoxef	2020/11/11	2020/12/2	21 days	*C. butyricum* Powders	2020/11/16	8 a.m. 3 p.m	2020/11/23	Yes
	Male	35 + 1	3	VD	2120	2020/11/22	latamoxef	2020/11/22	2020/12/6	14 days	*C. butyricum* Powders	2020/11/25	8 a.m. 3 p.m	2020/12/2	Yes
	Male	34 + 3	3	VD	1700	2020/12/1	latamoxef	2020/12/1	2020/12/19	18 days	*C. butyricum* Powders	2020/12/4	8 a.m. 3 p.m	2020/12/11	Yes

VD, vaginal delivery, CS, cesarean section. Daytime (8a.m. ~ 3p.m.), Nighttime (8p.m. ~ 3a.m.)

**Figure 6:**
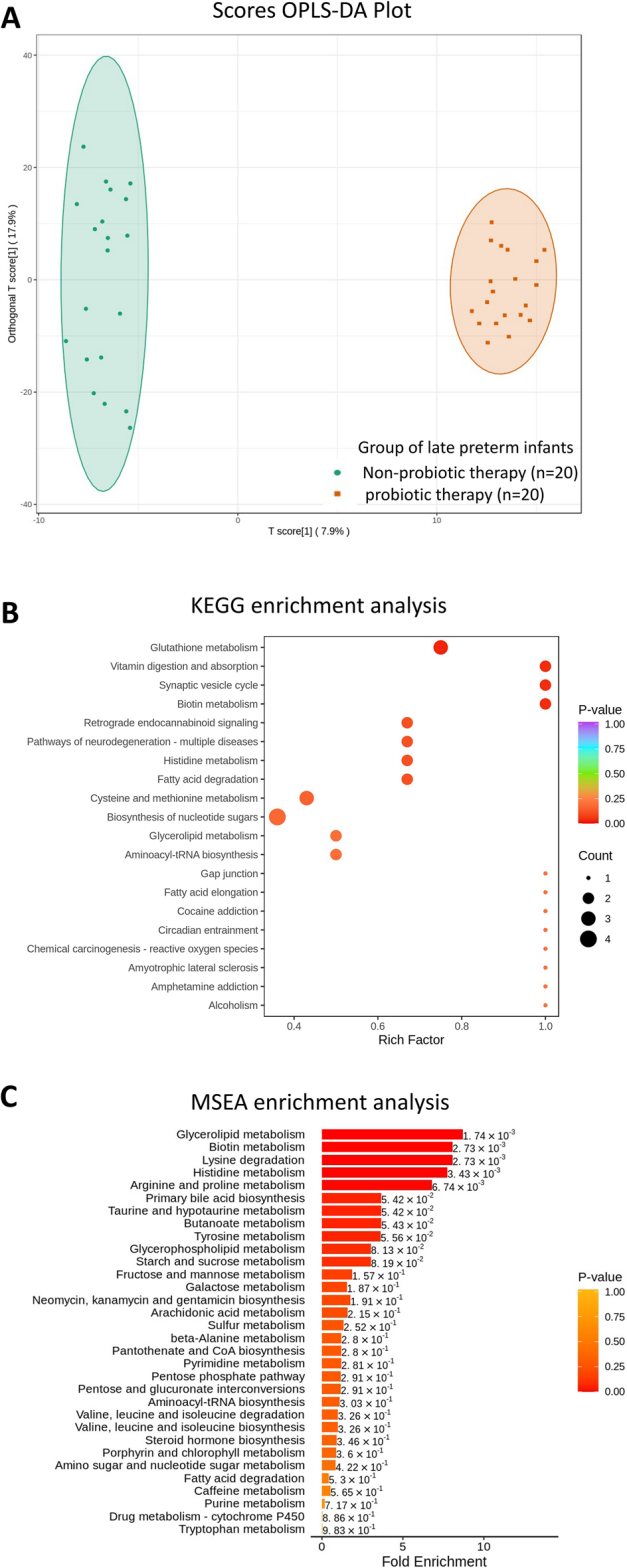
Fecal metabolome and metabolites were analyzed in late preterm infants with (n = 20) or without probiotic therapy (n = 20). (**A**) Scores OPLS-DA Plot indicated the similarity of metabolites between groups. (**B**) KEGG enrichment analysis. (**C**) MSEA enrichment analysis.

After that, Orthogonal Projections to Latent Structures Discriminant Analysis (OPLS-DA) was enrolled to analyze the principal component of metabolites. The general presence of metabolites was shown in Fig. 6 between the late preterm infants with (n = 20) or without (n = 20) probiotic therapy. The model of OPLS-DA illustrated the variations of metabolites between these two groups (Fig. 6A). Then, KEGG enrichment analysis and Metabolite Set Enrichment Analysis (MSEA) were employed to identify and interpret patterns of metabolite concentration changes. Intriguingly, the KEGG enrichment analysis showed that vitamin digestion and absorption, synaptic vesicle cycle, biotin metabolism (Group B vs. C, 0.1 rich factor) were significantly increased in the probiotic-treated group (Fig. 6B). MSEA also showed that a series of metabolism were significantly enriched in probiotic-treated infants including glycerolipid (Group B vs. C, 1.74 × 10^–threefold^ enrichment), biotin (Group B vs. C, 2.73 × 10^–threefold^ enrichment), lysine (Group B vs. C, 2.73 × 10^–threefold^ enrichment), and another metabolism (Fig. 6C), indicating the complex effects of probiotic therapy on glutathione metabolism and nutrients digestion and absorption in late preterm infants.

## Discussion

Compared with adults, infant gut microbes are typically less diverse, and unstable with marked individual variations^24–26^. It takes 2–3 years before the gut microbes become fully developed^3^. In our study, a high abundance of commensal intestinal bacteria was detected in full-term infants such as *E. coli* and *S. aureus*. The presence of Staphylococcus aureus in feces may be due to introduction of the bacteria into the infants while sucking breast milk^26^. However, decreases in the abundance of gut microbes (e.g. OTU6, *Bacteroidota*) were observed in the late preterm infants with dominant colonization of *Proteobacteria* and *Firmicutes* (Supplementary Fig. 1). Such decreased diversity was reported in several studies, which might be involved in diseases such as Type I diabetes and obesity^27, 28^. However, all the infants enrolled in this study grew in good conditions according to a 1-year telephone follow-up.

The abundance and diversity of gut microbes in infants can be altered by numerous factors, including maternal diet, human behaviors, environmental issues, genetic variations, and other conditions^29–33^. In this study, a minority of oral bacteria was detected in late preterm infants including *Veillonella parvula*, *Haemophilus influenzae*, *Rothia mucilaginosa,* and *Streptococcus sanguinis*, suggesting that human behaviors (e.g., breast breeding) could perform as a neglected pathway to influence the abundance and diversity of gut microbes in the infants^34^.

In addition, previous studies demonstrated the variants of gut microbes between full-term infants and late preterm infants. Preterm newborns had a lower proportion of *Bacteroidaceae* and *Bifidobacterium* in the first few months after birth compared to full-term infants^35^. The gut microbiota is impacted by perinatal antibiotics, especially intrapartum antimicrobial prophylaxis, as shown by an increase in Enterobacteriaceae family species in the neonates, which was consistent with our observations. However, full-term infants gradually established the colonization of *Lactobacillus*, indicating the potential role of probiotics in infant maturation^36^. Furthermore, Jia et al., also reported that gut microbes of preterm infants were gradually catching up with that of term infants 120 days after birth^37^. However, this study only included infants aged < 7 days and observed the gut microbes one week after taking probiotics. Further studies are needed to verify the long-term effects of probiotics.

Other issues to be noted in this study were the late preterm infants with mandatory antimicrobial consumption. Such latamoxef therapy might lead to the dominant colonization of *Enterococcus* in infant guts. It is common that preterm infants to receive early and frequent extended antimicrobial therapies. Antimicrobials such as meropenem, cefotaxime, and ticarcillin–clavulanate are reported with uniform effects on the reduction of species richness^38^.

*Clostridium butyricum* was a probiotic wildly used for protecting the human gut from various intestinal conditions, such as intestinal injuries and infections, irritable bowel syndrome, inflammatory bowel disease, and colorectal cancer^39^. A recent study showed that *C. butyricum* plays a crucial role in preventing *Clostridium difficile* proliferation and enterohemorrhagic *E. coli* infection^40, 41^. In this study, late preterm infants treated by *C. butyricum* from Group C showed a lower proportion of *Staphylococcus* spp., *Sphingomonas* spp. and *Pseudomonas* spp. (Fig. 3F), suggesting probiotics as a potential auxiliary therapy to help reduce the opportunistic pathogens in the gut. In addition, this study observed a significant decrease of evenness in *C. butyricum*-treated group, while there were no differences in community richness. This may be explained by the fact that the administration of probiotics did not considerably alter the total bacterial population; nonetheless, probiotics occupied the gut microbiota and significantly reduced species evenness^42^. This could account for the decline in the Shannon and Simpson indices, which coincided with the reduction of opportunistic pathogens such as *Staphylococcus aureus*, *Sphingomonas echinoides*, and *Pseudomonas putida* in this study.

Moreover, researchers found that antimicrobials given to mice at night significantly increased plasma concentrations of amoxicillin and sulfamethoxazole^43^. In this study, the persistence of *C. butyricum* was remarkably increased in the night-fed group. Meanwhile, *Enterococcus faecium* was observed to reduce in the night-fed group compared with the day-fed group. However, further studies are needed to support and elucidate the role and mechanisms by which probiotics impact the balance of gut microbes in infants.

Finally, a series of metabolism were significantly enriched in probiotic-treated infants including glycerolipid, biotin, and lysine in relation to the function of glutathione metabolism and nutrient digestion and absorption (Fig. 6). The glycerolipid (GL) cycle involved the continuous formation and hydrolysis of GL with simultaneous release of heat^44^. Recent studies evidenced the associations between GL cycle and diseases including obesity, type 2 diabetes, and cancer^44–46^. Previous study also observed an increase of glycerolipid in diet-induced obesity mice fed by probiotic *L. casei* LC-XCAL™^47^. These compounds were considerably higher in the feces of newborns in the probiotic-treated group in this study, indicating modest improvements in immunity and health in infants. Similarly, the metabolism of pyruvate, glycerolipid, propanoate, and fatty acid biosynthesis was found to be enriched in the probiotic groups in intestinal microbiome treated with *Lactobacillus plantarum* HNU082 and *Clostridium butyricum* MIYAIRI 588 as previously reported, indicating the functional shift toward increased carbohydrate metabolism in the intestinal microenvironment^48, 49^. However, further study is required to elucidate the associations between *C. butyricum* and certain metabolomic patterns in the gut.

There are some limitations to this study. This study only reflects the conditions of the infant gut microbes in East China. It is generally accepted that regional and environmental factors may influence gut microbial composition even more than human age and gender^50, 51^. This study focuses on Chinese late preterm one week after premature birth and full-term infants aged 38–40 weeks. Other factors such as ethnicity, geography, genetics, and parental lifestyle were not considered. In addition, the prevalence of microbial groups changed significantly with the age of the infant in the early life^52, 53^. The stool was collected within 24 h in Group A while feces were collected after one week probiotics intervention in Group C. This time of week may have an impact on the gut microbial composition, and studies need to further elucidate the effect of probiotics on the intestinal bacteria of infants with the same age.

In conclusion, this study reported the composition, abundance, and diversity of gut microbes in full-term and late-preterm infants in East China. Commensal intestinal bacteria were detected in these full-term infants whiles pathogenic *Enterococcus* species were dominant in late preterm group. Nevertheless, the consumption of *C. butyricum* was not able to restore the composition of gut microbes altered by antimicrobials to its normal level, although several opportunistic pathogens were found to decline significantly after probiotic therapy including *Staphylococcus aureus*, *Sphingomonas echinoides*, and *Pseudomonas putida*. Finally, a series of metabolism were significantly enriched in probiotic-treated infants including glycerolipid, biotin, and lysine, indicating the complex effects of probiotic therapy on glutathione metabolism and nutrient digestion and absorption in late preterm infants. Further studies are needed to support and elucidate the role of probiotic *C. butyricum* feeding in late preterm infants of mandatory antimicrobial treatment.

### Supplementary Information

Supplementary Information 1.

Supplementary Information 2.

Supplementary Information 3.

Supplementary Information 4.

Supplementary Information 5.

## Data Availability

The raw reads from this study are submitted to the China National Genomics Data Center under the link of https://bigd.big.ac.cn/gsa/browse/CRA012623.
