# Supplementary figures and images for: The metagenomic and metabolomic profile of the gut microbes in Chinese full-term and late preterm infants treated with Clostridium butyricum
Source: Sci Rep. 2023 Oct 31;13:18775. doi: 10.1038/s41598-023-45586-2 (PMC10618524; doi:10.1038/s41598-023-45586-2)

**A**

Barplot

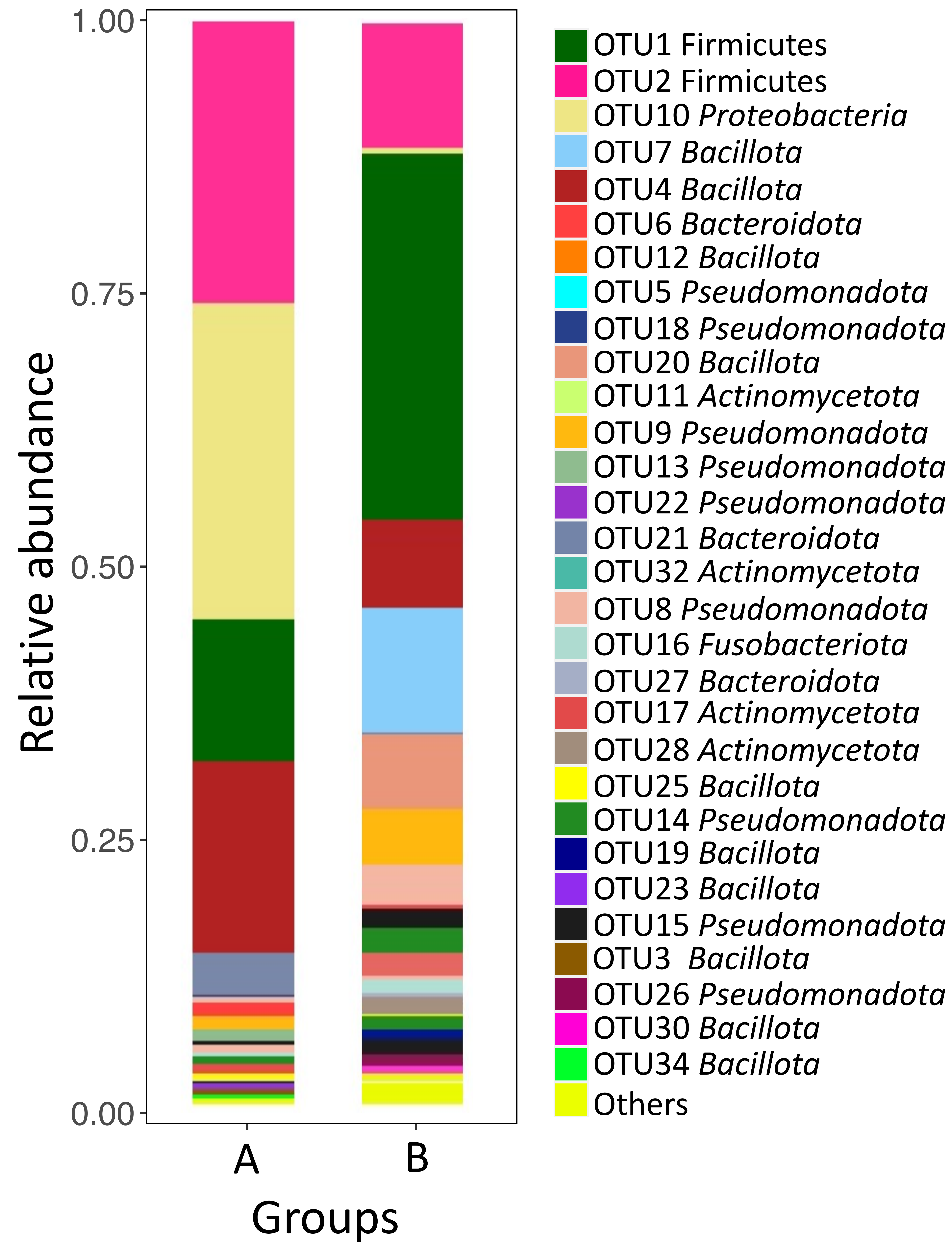**B**

Barplot

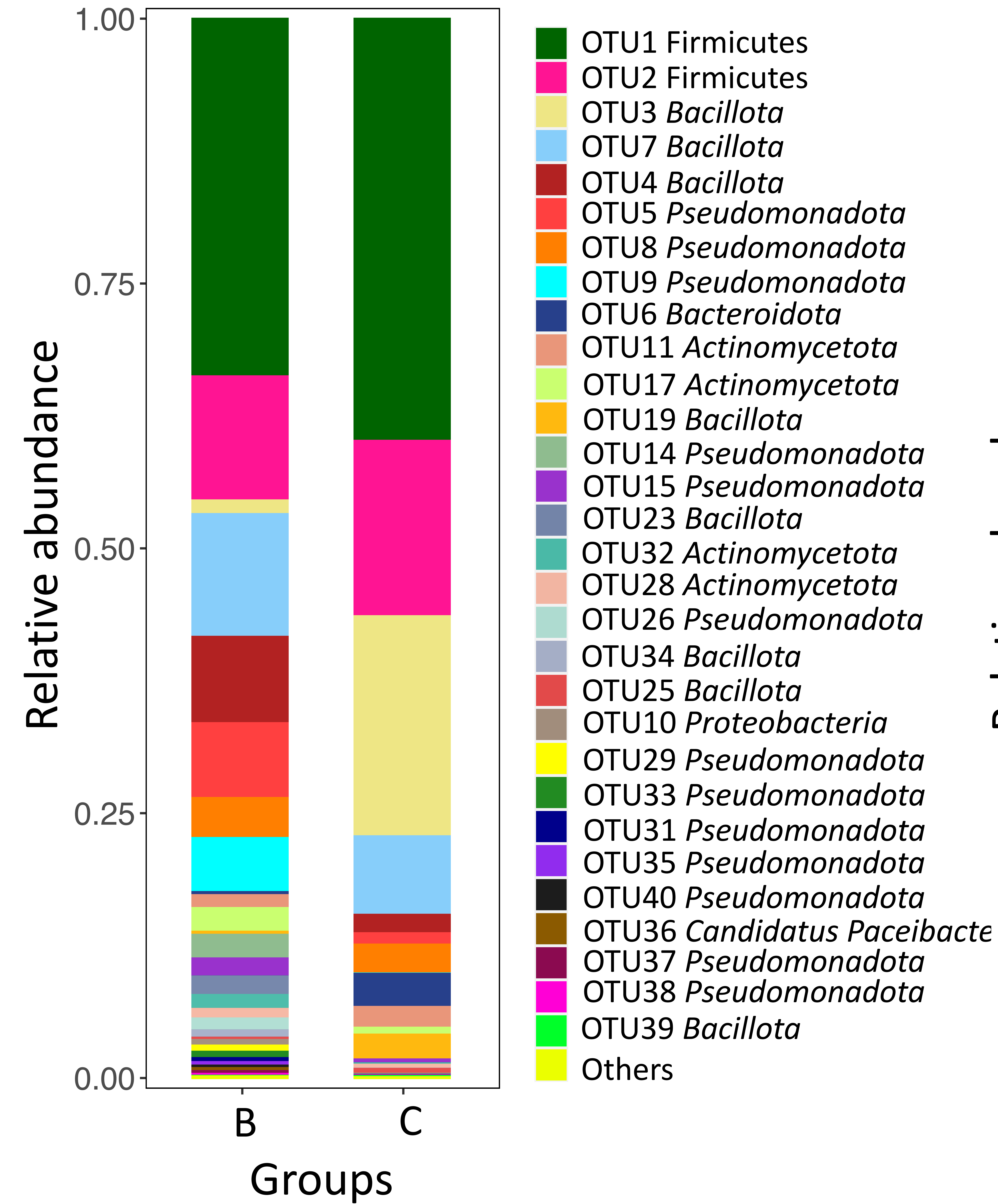**C**

Barplot

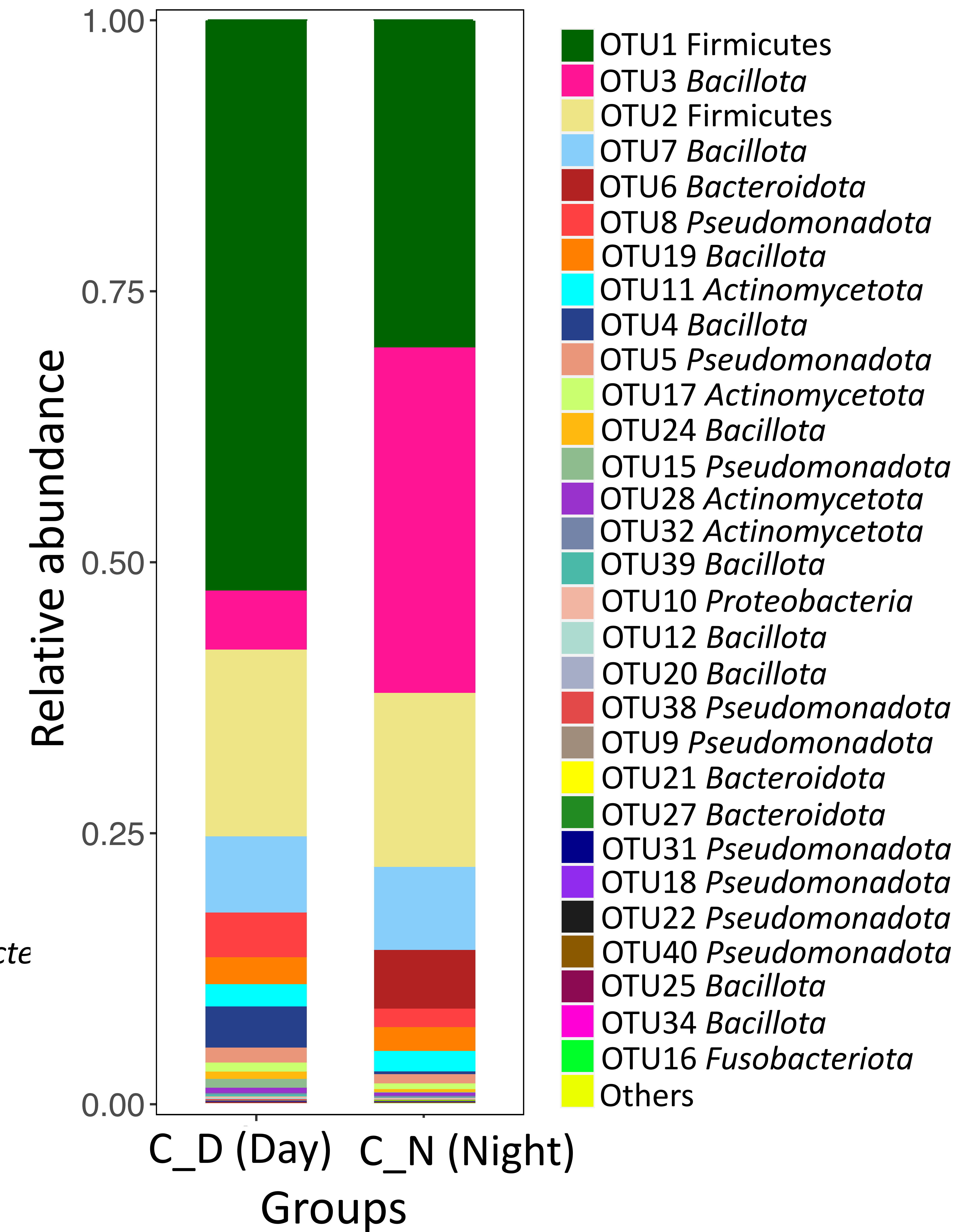

Supplement: Supplementary file 1 — Supplementary Information 1. [file 41598_2023_45586_MOESM1_ESM.pdf]
