# Supplementary material for: The metagenomic and metabolomic profile of the gut microbes in Chinese full-term and late preterm infants treated with Clostridium butyricum
Source: Sci Rep. 2023 Oct 31;13:18775. doi: 10.1038/s41598-023-45586-2 (PMC10618524; doi:10.1038/s41598-023-45586-2)
Supplement: Supplementary file 3 — Supplementary Information 3. [file 41598_2023_45586_MOESM3_ESM.docx]

**Supplementary materials**

**Supplementary Table 2.** The compound name of internal standard used in LC/MS analysis.

| Model | MWID | Chemical Name | m/z | RT | CV | chemical formula | Molecular mass | Model | Q1 | CAS |
| --- | --- | --- | --- | --- | --- | --- | --- | --- | --- | --- |
| neg | MWS5255 | [2H4]-Succinic acid | 121.0444 | 1.30 | 0.0110 | C4H2D4O4 | 122.0517 | M-H | 121.0444 | 14493-42-6 |
| neg | MWS4256 | L-Phenylalanine (2-13C,99%) | 164.0712 | 1.81 | 0.0144 | C9H11NO2 | 165.079 | M-H | 164.0712 | 167088-01-9 |
| neg | MWS2852 | Indole-3-carboxylic Acid-d5 | 165.0718 | 4.33 | 0.0071 | C9H2D5NO2 | 166.0791 | M-H | 165.0718 | IR-73457 |
| neg | MWS0923 | 2-Chlorophenylalanine | 198.0326 | 2.44 | 0.0132 | C9H10NClO2 | 199.04 | M-H | 198.0326 | 14091-11-3 |
| neg | MWS3171 | DL-3-Indolelactic acid (ILA) | 204.0666 | 4.11 | 0.0369 | C11H11NO3 | 205.0739 | M-H | 204.0666 | 832-97-3 |
| pos | MWS1777 | 3-Chloroaniline | 128.0262 | 5.70 | 0.0276 | C6H6ClN | 127.0189 | M+H | 128.0262 | 108-42-9 |
| pos | MWS4256 | L-Phenylalanine (2-13C,99%) | 166.0868 | 1.80 | 0.0147 | C9H11NO2 | 165.079 | M+H | 166.0868 | 167088-01-9 |
| pos | MWS2852 | Indole-3-carboxylic Acid-d5 | 167.0863 | 4.32 | 0.0226 | C9H2D5NO2 | 166.0791 | M+H | 167.0863 | IR-73457 |
| pos | MWS5078 | 4-fluoro-L-2-phenylglycine | 170.0617 | 1.19 | 0.0226 | C8H8FNO2 | 169.0539 | M+H | 170.0617 | 19883-57-9 |
| pos | MWS0923 | 2-Chlorophenylalanine | 200.0473 | 2.45 | 0.0200 | C9H10NClO2 | 199.04 | M+H | 200.0473 | 14091-11-3 |
